# Decitabine in patients with myelodysplastic syndromes: A multi‐center, open‐label, dose comparison trial

**DOI:** 10.1002/cam4.5922

**Published:** 2023-06-23

**Authors:** Hui Liu, Hao Jiang, Hongyan Tong, Ruixiang Xia, Linhua Yang, Hongguo Zhao, Jian Ouyang, Hai Bai, Hui Sun, Li Hou, Ming Jiang, Yun Zeng, Zhuogang Liu, Aibin Liang, Yinghua Xie, Kang Yu, Zhimin Zhai, Li Liu, Jinsong Jia, Rong Fu, Zonghong Shao

**Affiliations:** ^1^ Department of Hematology Tianjin Medical University General Hospital Tianjin China; ^2^ Institute of Hematology Peking University People's Hospital Beijing China; ^3^ Department of Hematology The First Affiliated Hospital, Zhejiang University School of Medicine Hangzhou China; ^4^ Department of Hematology The First Affiliated Hospital of Anhui Medical University Hefei China; ^5^ Department of Hematology The Second Hospital of Shanxi Medical University Taiyuan China; ^6^ Department of Hematology The Affiliated Hospital of Qingdao University Qingdao China; ^7^ Department of Hematology, Affiliated Drum Tower Hospital Medical School of Nanjing University Nanjing China; ^8^ Department of Hematology, Lanzhou General Hospital Lanzhou Military Area Lanzhou China; ^9^ Department of Hematology The First Affiliated Hospital of Zhengzhou University Zhengzhou China; ^10^ Department of Hematology, West China Hospital Sichuan University Chengdu China; ^11^ Department of Hematology The First Affiliated Hospital of Xinjiang Medical University Urumqi China; ^12^ Department of Hematology The First Affiliated Hospital of Kunming Medical University Kunming China; ^13^ Department of Hematology Shengjing Hospital of China Medical University Shenyang China; ^14^ Department of Hematology/Oncology TongJi Hospital of Tong Ji University Shanghai China; ^15^ Department of Hematology The Shanghai Fifth People's Hospital, Fudan University, Blood Disease Research Center, Fudan University Shanghai China; ^16^ Department of Hematology The First Affiliated Hospital of Wenzhou Medical University Wenzhou China; ^17^ Department of Hematology The Second Affiliated Hospital of Anhui Medical University Hefei China; ^18^ Department of Hematology, Tangdu Hospital PLA Air Force Military Medical University Xi'an Shanxi China

**Keywords:** complete remission, decitabine, hypomethylating agent, myelodysplastic syndromes, overall survival

## Abstract

**Background:**

The hypomethylating agent decitabine is the standard therapy for intermediate or high risk myelodysplastic syndrome (MDS).

**Methods:**

In this trial, 191 adult patients with intermediate/high risk MDS (IPSS score ≥ 0.5) randomly received decitabine using a standard regimen (20 mg/m^2^/day for 5 consecutive days; *n* = 94) or an extended regimen with lower daily dose (12 mg/m^2^/day for 8 consecutive days; *n* = 97) every 4 weeks, for a total of 4 cycles.

**Results:**

The median follow‐up was 14 months (range 2–36). The primary end point of overall response rate in the intent‐to‐treat analysis was 41.5% and 38.1% in the standard and extended dosing arms, respectively (*p* = 0.660). Complete remission and marrow complete remission also did not differ between the two arms. Cytopenia was the most frequent adverse event (76.4%). The median duration of neutropenia per cycle did not differ between the two arms during the first two cycles, but significantly shorter in the extended dosing arm in the third cycle (8.5 vs. 15.5 days, *p* = 0.049) and in the fourth cycle (8 vs. 14 days, *p* = 0.294).

**Conclusion:**

The 5‐day 20‐mg/m^2^/day and 8‐day 12‐mg/m^2^/day decitabine regimens have similar efficacy and safety in patients with intermediate or high risk MDS.

## Graphical abstract

In this trial, 191 adult patients with intermediate‐/high‐risk MDS (IPSS score = 0.5) randomly received decitabine using a standard regimen (20 mg/m^2^/day for 5 consecutive days; *n* = 94) or an extended regimen with lower daily dose (12 mg/m^2^/day for 8 consecutive days; *n* = 97) every 4 weeks, for a total of four cycles. We found that the 5‐day 20‐mg/m^2^/day and 8‐day 12‐mg/m^2^/day decitabine regimens have similar efficacy and safety in patients with intermediate‐ or high‐risk MDS.

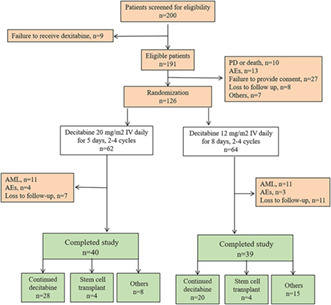

## 1 INTRODUCTION

Myelodysplastic syndrome (MDS) is a heterogeneous group of acquired clonal hematopoietic progenitor cell diseases characterized by ineffective hematopoiesis, cytopenia, and leukemic transformation.^1^, ^2^ Epigenetic changes, most notably aberrant DNA hypermethylation, are implicated in the pathogenesis and leukemic transformation of MDS.^3^, ^4^ The DNA hypomethylating agent decitabine is the standard therapy for higher risk MDS patients,^5^, ^6^ but the dosing regimen has been evolving, with a general trend for shorter courses but increasing dosage in each course.^7^, ^8^, ^9^, ^10^, ^11^, ^12^, ^13^

Decitabine was launched in China in 2009 without clinical trials. A subsequent phase 3b trial in 135 Chinese patients with de novo or secondary MDS showed 29.4% overall response rate (ORR) for 3‐h infusion at 15 mg/m^2^ every 8 h for 3 consecutive days/cycle/6 weeks and 25.5% ORR for 1‐h infusion at 20 mg/m^2^ once daily on days 1–5/cycle/4 weeks.^10^ A recent retrospective study of 13 Chinese patients with de novo MDS showed 69.2% ORR with intravenous decitabine at 6 mg/m^2^ per day for 7 days, repeated every 4 weeks.^14^ Up to date, there has been no clinical trials of decitabine in Chinese patients with intermediate‐ or high‐risk de novo MDS. We conducted a multicenter, open‐label, dose comparison trial to compare the efficacy and safety of two decitabine dosing regimens (20 mg/m^2^/day for 5 consecutive days vs. 12 mg/m^2^/day for 8 consecutive days, every 4 weeks) in Chinese patients with intermediate‐ or high‐risk de novo MDS.

## 2 METHODS

### 2.1 Patients

Adult (≥18 years) patients with intermediate‐ or high‐risk MDS (an IPSS score ≥ 0.5 and an ECOG performance status score at 0–2) were eligible. MDS was diagnosed according to the 2008 World Health Organization (WHO) classification, WHO classification for RCUD, RARS and transfusion‐dependent RCMD. Exclusion criteria included: (1) previous acute myeloid leukemia (AML); (2) other malignancies within 12 months; (3) prior therapy with azacitidine or decitabine; and (4) active or uncontrolled infection.

Trial protocol adhered to the SPIRIT statement^15^ and was approved by the ethics committees of all participating institutions (Appendix A). Written informed consent was obtained before enrollment. The trial is registered with ClinicalTrials.gov (NCT02013102) and was conducted in accordance with the Declaration of Helsinki.

### 2.2 Intervention

Patients were randomized at a 1:1 ratio to receive intravenous decitabine at 20 mg/m^2^/day for 5 days (standard dosing arm) or at 12 mg/m^2^/day for 8 days (extended dosing arm) every 4 weeks for a total of four cycles. Treatment was discontinued upon disease progression, severe infection, major bleeding, or severe myelosuppression. All patients received best supportive care.

### 2.3 Efficacy evaluation

The primary endpoint of ORR, as defined by the modified International Working Group 2006 (IWG 2006) criteria,^16^ was compared in both the intent‐to‐treat (ITT) and per‐protocol population. ORR included complete response (CR), marrow CR (mCR), and partial response (PR). Secondary efficacy endpoints, including CR, mCR, PR, hematologic improvement (HI), cytogenetic response, and transfusion requirements, were analyzed in the per‐protocol population. Routine blood examination was performed every week. Bone marrow (BM) was examined every two cycles. Both overall survival (OS) and progression‐free survival (PFS) were calculated from the day when therapy was initiated.

### 2.4 Safety evaluation

Safety was evaluated in all patients who received at least one dose of the investigational drug using the CTCAE version 4.0, and coded to a preferred term using the Medical Dictionary for Regulatory Activities (MedDRA). Severe adverse events (SAEs) were defined as any AE that resulted in death, was life‐threatening, required hospitalization, prolonged hospitalization, caused significant or persistent disability or incapacity, or birth defects. All patients were followed until recovery from any treatment‐emergent AEs (TEAEs).

### 2.5 Statistical analysis

Statistical analyses were conducted using SAS 9.4. Continuous variables were compared with Student's *t*‐test or Wilcoxon signed sum test. Categorical variables were analyzed with *χ* ^2^ test or Fisher's exact test, as appropriate. Changes from baseline were compared using analyses of variance (ANOVA) or rank‐sum test. The ITT population included all patients who received at least one dose of the study drug and had a baseline assessment and at least one post‐baseline assessment. The per‐protocol population included patients who completed at least two treatment cycles as planned and underwent efficacy evaluation. *p* ≤ 0.05 (two‐sided) was considered statistically significant and 95% confidence interval (CI) was used to describe the results.

## 3 RESULTS

### 3.1 Demographic and baseline characteristics

**FIGURE 1:**
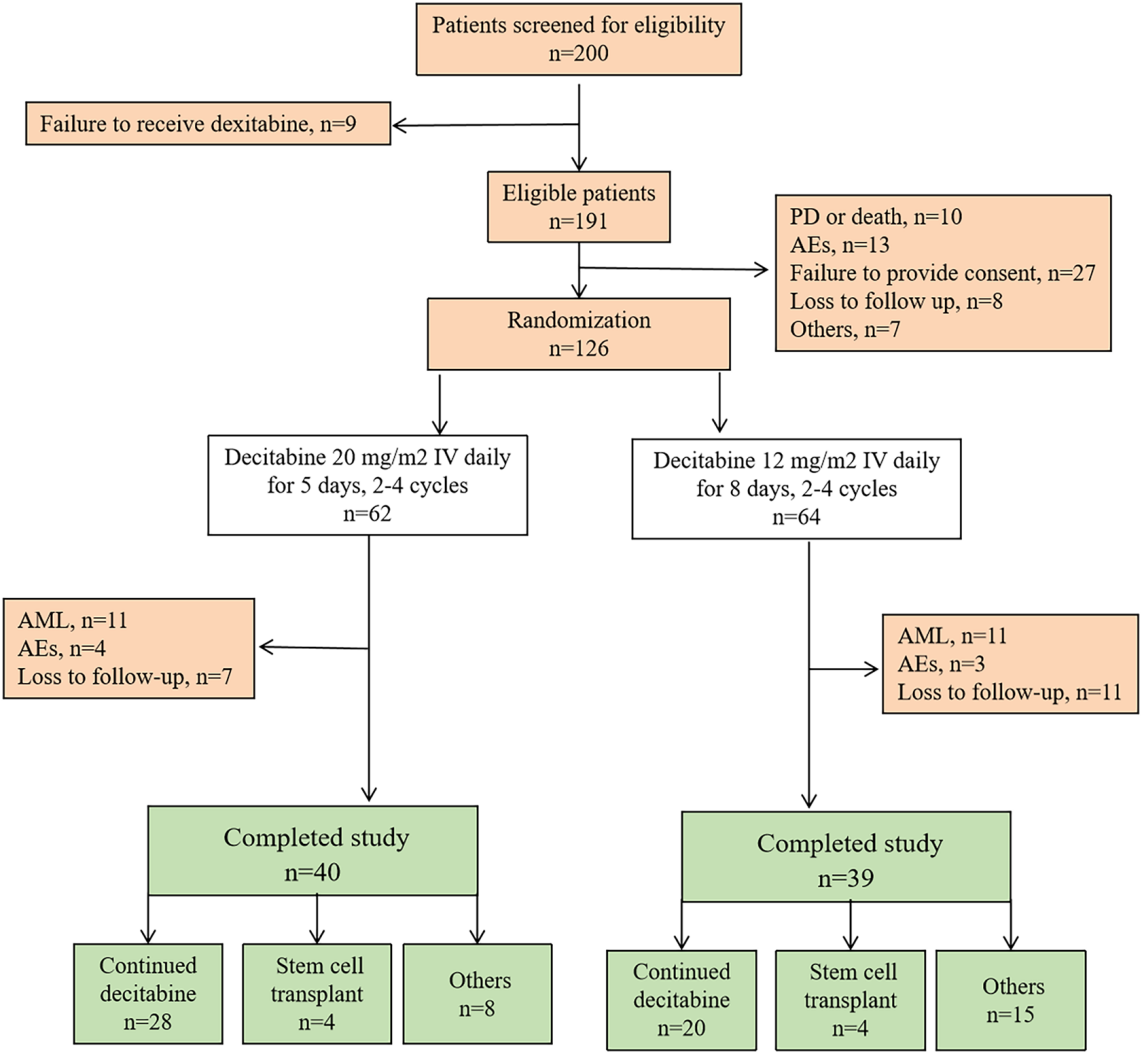
The study flowchart. AML, acute myeloid leukemia; IV, intravenous; PD, progressive disease.

The study flowchart is shown in Figure 1. Demographic and baseline characteristics of the patients are provided in Table 1. A total of 200 patients with newly diagnosed MDS from 18 hospitals in China were screened for eligibility; 191 were randomized (94 and 97 in the standard and extended dosing arms, respectively). The median age was 57 years (range, 27–84), and 62.8% of the patients were men. The risk was intermediate‐1 in 38.7%, intermediate‐2 in 40.3% and high in 20.4% of the patients. Sixty‐five patients did not receive at least two cycles of treatment, leaving 126 patients (62 and 64 patients in the two arms, respectively) in the per‐protocol analysis.

**TABLE 1:** Patient demographic and baseline characteristics.

Characteristics	Subgroup	Standard dosing arm (*n* = 94)	Extended dosing arm (*n* = 97)	*p* value
Age (years)	Median (range)	57.00 (20–79)	57.00 (27–84)	0.9551
	>65	21 (22.34%)	22 (22.68%)	
Gender	Male	62 (65.96%)	60 (61.86%)	0.5552
	Female	32 (34.04%)	37 (38.14%)	
ECOG performance status score	0	18 (19.15%)	22 (22.68%)	0.6201
	1	62 (65.96%)	59 (60.82%)	
	2	14 (14.89%)	16 (16.49%)	
MDS subtype (WHO classification)	RCUD	3 (3.19%)	1 (1.03%)	0.3989^a^
	RARS	2 (2.13%)	4 (4.12%)	
	RCMD	20 (21.28%)	18 (18.56%)	
	RAEB‐1	33 (35.11%)	26 (26.80%)	
	RAEB‐2	36 (38.30%)	45 (46.39%)	
	5q‐	0	2 (2.06%)	
	Lost	0	1 (1.03%)	
IPSS	Intermediate‐1	39 (41.49%)	35 (36.08%)	0.3960
	Intermediate‐2	37 (39.36%)	40 (41.24%)	
	High risk	18 (19.15%)	21 (21.65%)	
	Lost	0	1 (1.03%)	
Comorbidities	No	39 (41.49%)	37 (38.14%)	0.5164
	Yes	54 (57.45%)	60 (61.86%)	
	Lost	1 (1.06%)	0	

*Note*: MDS subtype was based on the WHO Classification.

Abbreviations: ECOG, Eastern Cooperative Oncology Group; IPSS, International Prognostic Scoring System; MDS, myelodysplastic syndrome; RAEB, refractory anemia with excess blasts; RARS, refractory anemia with ring sideroblasts; RCMD, refractory anemia with multilineage dysplasia; RCUD, refractory cytopenia with unilineage dysplasia.

### 3.2 Treatment characteristics

The median treatment duration was 70 days (range 28–112) in the standard dosing arm and 56 days (range 28–112) in the extended dosing arm. The median number of treatment cycles was 2.5 (range, 1–4) and 2.0 (range, 1–4), respectively. The median total dosage was 170.5 mg (range, 56–218.5) and 166.2 mg (range, 90.2–211), respectively. Decitabine dose reduction occurred in 2 (2.1%) patients in each arm. Dose interruption was reported in 2 (2.1%) and 1 (1.0%), respectively.

### 3.3 Efficacy endpoints

ORR did not differ between the two arms, either in the ITT analysis (41.5% in the standard dosing arm vs. 38.1% in the extended dosing arm; *p* = 0.660) or in the per‐protocol analysis (62.9% vs. 57.8%; *p* = 0.589; Table 2). The two arms did not differ in CR (27.4% vs. 21.9%, *p* = 0.538), mCR (25.8% vs. 31.3%, *p* = 0.557), PR (9.7% vs. 4.7%, *p* = 0.361), cytogenetic response (8.1% vs. 6.3%, *p* = 0.552), or blood transfusion (38.7% vs. 53.1%, *p* = 0.112).

**TABLE 2:** Efficacy endpoints.

	Standard dosing arm	Extended dosing arm	*p* value
Intent‐to‐treat analysis (*N* = 94 and 97)			
ORR	41.5%	38.1%	0.660
CR	18.1%	14.4%	0.558
mCR	17%	20.6%	0.581
PR	6.4%	3.1%	0.326
HI	3.2%	1%	0.363
Cytogenetic response	5.3%	4.1%	0.556
Per‐protocol analysis (*N* = 62 and 64)			
ORR	62.9%	57.8%	0.589
CR	27.4%	21.9%	0.538
mCR	25.8%	31.3%	0.557
PR	9.7%	4.7%	0.320
HI	4.8%	1.6%	0.361
Cytogenetic response	8.1%	6.3%	0.552

*Note*: Data are shown as *n* (%).

Abbreviations: CR, complete response; HI, hematological improvement; mCR, marrow complete response; ORR, objective response rate; PR, partial response.

Subgroup analysis stratified by age (cutoff at 65 years) failed to show significant difference in the ORR between the two arms in either subgroup (Table 3). Subgroup analyses based on risk or WHO classification subtype also failed to show significant difference in the ORR between the two arms.

**TABLE 3:**
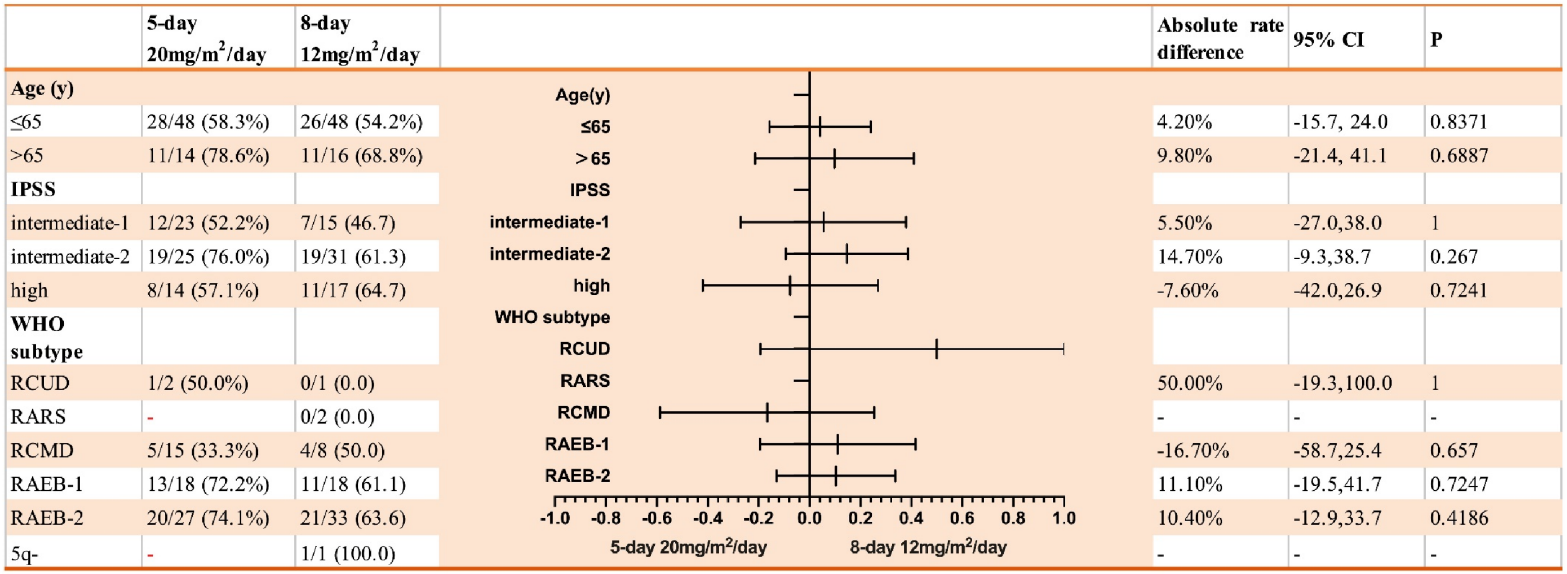
Subgroups analysis of overall response (per‐protocal set).

*Note*: Data are event number (overall response)/patient number.

Abbreviations: IPSS, International Prognostic Scoring System; RAEB, refractory anemia with excess blasts; RARS, refractory anemia with ringed sideroblasts; RCMD, refractory cytopenia with multilineage dysplasia; RCUD, refractory cytopenia with unilineage dysplasia.

### 3.4 Survival

The median follow‐up was 32 months (range 2–70). Twenty (25/126, 19.8%) patients were lost to follow‐up. Thirty‐eight patients died, 23 (60.5%) due to AML progression, 9 (23.7%) due to infections, 5 (13.2%) from sudden cardiac death and 1 (2.6%) because of bleeding. The median PFS was 13 months (range 2–33) in the standard dosing arm and 15 months (range 2–38) in the extended dosing arm (*p* = 0.651). The two arms had similar 6‐month OS (81.8% vs. 84.9%, *p* = 0.832), 12‐month OS (68.2% vs. 65.9%, *p* = 0.766), and 24‐month OS (51.4% vs. 53.1%, *p* = 0.943).

### 3.5 Safety

The two arms did not differ in AEs (94.7% vs. 93.8%, *p* = 0.797), SAEs (6.4% vs. 11.3%, *p* = 0.229), adverse drug reactions (ADRs; 85.1% vs. 84.5%, *p* = 0.913) and severe ADRs (3.2% vs. 4.1%, *p* value is not significant). The rate of hematologic TEAEs also did not differ between the two arms (77.66% vs. 75.26%, *p* = 0.875). The most common grade 3/4 hematologic TEAEs included anemia (40.4%), thrombocytopenia (38.3%), and leucopenia (25.5%) in the standard dosing arm, and thrombocytopenia (32.0%), anemia (28.9%), and granulocytopenia (20.6%) in the extended dosing arm (Table 4). The median duration of neutropenia per cycle did not differ between the two arms in the first two cycles, but was significantly shorter in the extended dosing arm in the third cycle (8.5 vs. 15.5 days, *p* = 0.049) and in the fourth cycle (8 vs. 14 days, *p* = 0.294).

**TABLE 4:** Adverse events in the safety set.

	Arm I	Arm II	*p* value
	*N* (%)	*N* (%)	
Grade 3 and above hematologic TEAEs			
Neutropenia	24 (25.5)	20 (20.6)	0.427
Leucopenia	22 (23.4)	19 (19.6)	0.606
Thrombocytopenia	36 (38.3)	30 (30.9)	0.298
Anemia	38 (40.4)	28 (28.9)	0.102
Grade 3 and above non‐hematologic TEAEs			
Hepatobiliary abnormalities	3 (3.2)	5 (45.2)	<0.001
Infections	11 (11.7)	13 (13.4)	0.831
Gastrointestinal abnormalities	1 (1.1)	5 (5.2)	0.212
Nervous system	2 (2.1)	2 (2.1)	1
Heart	4 (4.3)	2 (2.1)	0.683
Skin and subcutaneous tissue	0 (0)	3 (3.1)	0.246
Drug delivery site	5 (5.3)	10 (10.3)	0.179
Skeletal muscle	0 (0)	0 (0)	1
Respiratory tract and chest	2 (2.1)	5 (5.2)	0.445
Vessels	1 (1.1)	1 (1.0)	0.999
Eyes	0 (0)	0 (0)	1
Nutrition and metabolism	2 (2.1)	1 (1.0)	0.999
Kidney and urethra	0 (0)	0 (0)	1
Dose modifications as a result of TEAEs			
Decitabine discontinuation	5 (5.3)	13 (13.4)	0.048
Decitabine reduction	1 (1.1)	3 (3.1)	0.621
Decitabine interruption	4 (4.3)	10 (10.3)	0.164

Abbreviation: TEAE, treatment emergent adverse event.

The two arms did not differ in nonhematologic TEAEs, including hepatobiliary abnormalities (21.28% vs. 26.8%), infections (32.98% vs. 36.08%), gastrointestinal abnormalities (29.79% vs. 41.24%), abnormalities in the nervous system (18.09% vs. 20.62%), heart (11.7% vs. 14.43%), and skin and subcutaneous tissues (17.02% vs. 23.71%), reaction in injection site (60.64% vs. 65.98%), abnormalities in the skeletal muscles (7.45% vs. 6.19%), the respiratory tract and chest (23.4% vs. 36.08%), blood vessels (5.32% vs. 3.09%), the eyes (4.26% vs. 1.03%), nutrition and metabolism (23.4% vs. 18.56%), and kidneys (3.19% vs. 1.03%) (*p* > 0.05 for all). The three most common grade 3 and 4 nonhematologic TEAEs were infections (11.7%), injection site pain (5.3%), and heart disorder (4.3%) and in the standard dosing arm, infection (13.4%), injection site pain (10.3%), respiratory tract and chest disorder (5.2%) and gastrointestinal abnormalities (5.2%) in the extended dosing arm (Table 4). No previously unreported AEs were observed.

## 4 DISCUSSION

The trial showed similar efficacy measures (including ORR, CR, mCR, and PFS) and safety profiles in the two arms, suggesting that either the 5‐day 20‐mg/m^2^/day or 8‐day 12‐mg/m^2^/day decitabine regimen is appropriate for use in Chinese patients with intermediate‐ or high‐risk MDS. There seemed to be marginal benefit in the duration of neutropenia with the extended dosing regimen.

Decitabine produces distinct effects at different dosages: it inhibits cell proliferation by irreversibly blocking DNA synthesis at high doses and blocks hypermethylation and consequently re‐expression of tumor suppressor genes at low doses.^17^, ^18^ Low dose decitabine (15 mg/m^2^, IV, over 3 h, every 8 h, 3 d, repeated every 6 weeks) was initially recommended for MDS, but was discontinued due to severe hematologic and nonhematologic toxicities. In a meta‐analysis of 1378 patients (15 studies), 100 mg/m^2^/course decitabine regimen had higher CR rate than the 135 mg/m^2^/course regimen, and higher ORR than the 60–75 mg/m^2^/course regimen.^19^ Currently, the recommended standard protocol in MDS patients is 20 mg/m^2^/day for 5 consecutive days, every 4 weeks. Several previous studies showed approximately 50% ORR, with low treatment‐emergent mortality.^20^, ^21^, ^22^, ^23^

In this trial, the median duration of neutropenia was shorter in the extended dosing arm in the third cycle (8.5 vs. 15.5 days) and in the fourth cycle (8 vs. 14 days), but not in the first two cycles. Such a potential benefit with the 8‐day 12‐mg/m^2^/day regimen requires verification in future studies. In a previous retrospective analysis,^24^ patients with dose modifications had a significantly higher ORR versus those without, suggesting better treatment effects with extended period of decitabine exposure.

The median age of MDS at the diagnosis is about 70 years, and many patients have comorbid conditions that could influence treatment decisions and prognosis. In this trial, the ORR was 78.6% and 68.8% in the two arms in patients >65 years of age versus 58.3% and 54.2% in younger patients, indicating that decitabine is more effective and safer in elderly Chinese patients.

About 70% of the patients in this trial had higher risk MDS, for which hypomethylating agents are the best option. Kantarjian et al. compared low intensity decitabine therapy with intensive chemotherapy in patients with higher risk MDS and found significant survival advantage with decitabine.^25^ In a phase 3 study by the EORTC Leukemia Cooperative Group and German MDS Study Group, decitabine prolonged PFS in high‐risk MDS patients with complex karyotypes harboring two or more autosomal monosomies.^26^ Another clinical trial showed that, in comparison to traditional chemotherapy, decitabine followed by low‐dose idarubicin plus cytarabine could reduce the rate of leukemic transformation in high‐risk myeloid neoplasms.^27^

Consistent with previous data,^8^ the median PFS for the entire patient cohort in the current study was 12 months, and 22 patients progressed to AML during the 14‐month follow‐up. Of note, decitabine resistance has been associated with more enriched somatic mutations, including mutations in *TP53*, *GATA2*, *KRAS*, *RUNX1*, *STAG2*, *ASXL1*, *ZRSR2*, and *TET2*.^28^, ^29^, ^30^, ^31^ Treatment options for such conditions include intensive chemotherapy (mainly based on anthracycline‐cytarabine combinations), allogeneic stem cell transplantation and targeted therapies (such as venetoclax, IDH1 or IDH2 inhibitors).^32^ Novel treatments under development include telomerase inhibitors and CTLA‐4 inhibitors.^33^, ^34^, ^35^, ^36^, ^37^

With the progress of detection technology and in‐depth study of pathogenesis, the treatment of MDS has made great progress. For the low‐risk group MDS, EPO, Eltrombopag, lenalidomide, Luspatercept, and iron removal treatment showed some efficacy. For the high‐risk group MDS, HMA treatment is one of the current standard treatments, but there are still unmet medical needs. Some new targeted drugs have been used for the treatment of high‐risk MDS, such as IDH1 inhibitor,^38^ Bcl2 inhibitor,^39^ XPO1 inhibitor,^40^ anti‐CD47 antibody,^41^ anti‐PD‐1antibody,^42^ et al., combined with azacytidine have shown good efficacy. Hematopoietic stem cell transplantation is the only curable method, while it still faces some problems, such as the selection of donors, the necessity of HMA bridging, the improvement of pretreatment scheme, the chimerism rate and implantation and MRD detection in the process of transplantation, and the “preemptive” treatment to prevent recurrence.

The rate of hematologic TEAEs in our study was practically identical in the two arms: 77.66% in the standard dosing arm and 75.26% in the extended dosing arm. Consistent with previous trials^43^, ^44^ and meta‐analyses,^45^, ^46^ the rate of grade 3/4 hematologic TEAEs were generally comparable between the two arms, but the median duration of neutropenia per cycle seemed to be shorter in the extended dosing arm in the third and fourth cycles.

This study has several limitations. First, 53 patients (19 and 34 in the two arms, respectively) did not complete four cycles of treatment as initially planned. Second, treatment after four cycles was not uniform, and could introduce bias to OS data. Future studies with larger sample size, longer follow‐up, and more meticulous data collection are needed. In adult Chinese patients with intermediate‐ or high‐risk MDS, decitabine is equally effective when given on an 8‐day 12‐mg/m^2^/day versus 5‐day 20‐mg/m^2^/day decitabine regimen, with generally comparable safety profile.

## AUTHOR CONTRIBUTIONS

**Hui Liu:** Conceptualization (equal); data curation (lead); writing – original draft (lead); writing – review and editing (equal). **Hao Jiang:** Conceptualization (lead); data curation (equal); writing – review and editing (equal). **Hongyan Tong:** Conceptualization (equal); data curation (equal); writing – review and editing (equal). **Ruixiang Xia:** Conceptualization (equal); data curation (equal); writing – review and editing (equal). **Linhua Yang:** Conceptualization (equal); data curation (equal); writing – review and editing (equal). **Hongguo Zhao:** Conceptualization (equal); data curation (equal); writing – review and editing (equal). **Jian Ouyang:** Conceptualization (equal); data curation (equal); writing – review and editing (equal). **Hai Bai:** Conceptualization (equal); data curation (equal); writing – review and editing (equal). **Hui Sun:** Conceptualization (equal); data curation (equal); writing – review and editing (equal). **Li Hou:** Conceptualization (equal); data curation (equal); writing – review and editing (equal). **Ming Jiang:** Conceptualization (equal); data curation (equal); writing – review and editing (equal). **Yun Zeng:** Conceptualization (equal); data curation (equal); writing – review and editing (equal). **Zhuogang Liu:** Conceptualization (equal); data curation (equal); writing – review and editing (equal). **Aibin Liang:** Conceptualization (equal); data curation (equal); writing – review and editing (equal). **Yinghua Xie:** Conceptualization (equal); data curation (equal); writing – review and editing (equal). **Kang Yu:** Conceptualization (equal); data curation (equal); writing – review and editing (equal). **Zhimin Zhai:** Conceptualization (equal); data curation (equal); writing – review and editing (equal). **Li Liu:** Conceptualization (equal); data curation (equal); writing – review and editing (equal). **Jinsong Jia:** Conceptualization (equal); data curation (equal); writing – review and editing (equal). **Rong Fu:** Conceptualization (equal); data curation (equal); writing – review and editing (equal). **Zonghong Shao:** Conceptualization (equal); data curation (equal); funding acquisition (lead); project administration (lead); writing – review and editing (lead).

## CONFLICT OF INTEREST STATEMENT

The authors confirm that there are no conflicts of interest.

## Data Availability

The datasets used and/or analyzed during the current study are available from the corresponding author on request.

## APPENDIX A

1. Tianjin Medical University General Hospital, Tianjin, China.

2. Peking University People's Hospital, Beijing, China.

3. The First Affiliated Hospital, Zhejiang University School of Medicine, Hangzhou, China.

4. The First Affiliated Hospital of Anhui Medical University, Hefei, China.

5. The Second Hospital of Shanxi Medical University, Taiyuan, China.

6. The Affiliated Hospital of Qingdao University, Qingdao, China.

7. Affiliated Drum Tower Hospital, Medical School of Nanjing University, Nanjing, China.

8. Lanzhou General Hospital, Lanzhou Military Area, Lanzhou, China.

9. The First Affiliated Hospital of Zhengzhou University, Zhengzhou, China.

10. West China Hospital, Sichuan University, Chengdu, China.

11. The First Affiliated Hospital of Xinjiang Medical University, Urumqi, China.

12. The First Affiliated Hospital of Kunming Medical University, Kunming, China.

13. Shengjing Hospital of China Medical University, Shenyang, China.

14. TongJi Hospital of Tong Ji University, Shanghai, China.

15. the Shanghai Fifth People's Hospital, Fudan University, Blood Disease Research Center, Fudan University, Shanghai, China.

16. The First Affiliated Hospital of Wenzhou Medical University, Wenzhou, China.

17. The Second Affiliated Hospital of Anhui Medical University, Hefei, China.

18. Tangdu Hospital, PLA Air Force Military Medical University, Shaanxi, China.
